# Gross’ Pathological Anatomy

**Published:** 1840-01

**Authors:** 


### GROSS' PATHOLOGICAL ANATOMY.

Dr. Gross, professor of *General and Pathological Anatomy and Physiology*, in the late Medical Department of the Cincinnati College, is about to appear as the author of a system of Pathological Anatomy, in two volumes, illustrated with numerous colored plates. It was issued from the Boston press in the month of November, but has not reached us. Our knowledge, however, of the eminent qualifications of the Author, inspires us with confidence in his work, which we have no doubt will present all the important facts, in this new and interesting branch of Medical Science, down to the present time; and it is creditable to Western Medicine, that it should have given to the profession the first separate and systematic treatise on morbid anatomy, in the United States. When received we shall make it the subject of a more extended notice. D.

